# Supplementary material for: Implantable photonic neural probes for light-sheet fluorescence brain imaging
Source: Neurophotonics. 2021 Apr 19;8(2):025003. doi: 10.1117/1.NPh.8.2.025003 (PMC8059764; doi:10.1117/1.NPh.8.2.025003)
Supplement: Supplementary file 1 [file NPh_008_025003_SD001.pdf]

Implantable photonic neural probes for light-sheet fluorescence brain imaging: supplementary materials

Wesley D. Sacher[1,2,3,4,*], Fu-Der Chen[3,†], Homeira Moradi-Chameh[5,†], Xianshu Luo[6,†], Anton Fomenko[5], Prajay Shah[5], Thomas Lordello[3], Xinyu Liu[1], Ilan Felts Almog[3], John N. Straguzzi[4], Trevor M. Fowler[1], Youngho Jung[3,4], Ting Hu[7], Junho Jeong[3], Andres M. Lozano[5,8], Patrick Guo-Qiang Lo[6], Taufik A. Valiante[5,8,9,3], Laurent C. Moreaux[1], Joyce K. S. Poon[3,4], Michael L. Roukes[1,2*]

[1] *Division of Physics, Mathematics, and Astronomy, California Institute of Technology, Pasadena, California 91125, USA*

[2] *Kavli Nanoscience Institute, California Institute of Technology, Pasadena, California 91125, USA*

[3] *Department of Electrical and Computer Engineering, University of Toronto, 10 King's College Rd., Toronto, Ontario M5S 3G4, Canada*

[4] *Max Planck Institute of Microstructure Physics, Weinberg 2, 06120, Halle, Germany*

[5] *Krembil Research Institute, Division of Clinical and Computational Neuroscience, University Health Network, Toronto, Ontario, Canada*

[6] *Advanced Micro Foundry Pte Ltd, 11 Science Park Road, Singapore Science Park II, 117685, Singapore*

[7] *Institute of Microelectronics, Agency for Science Technology and Research (A*STAR), 2 Fusionopolis Way, #08-02, Innovis, 138634, Singapore*

[8] *Division of Neurosurgery, Department of Surgery, Toronto Western Hospital, University of Toronto, Toronto, Ontario, Canada*

[9] *Institute of Biomaterials and Biomedical Engineering, University of Toronto, Toronto, Ontario, Canada*

[*] *Corresponding authors: wesley.sacher@mpi-halle.mpg.de, roukes@caltech.edu*

[†] *Equal contribution*

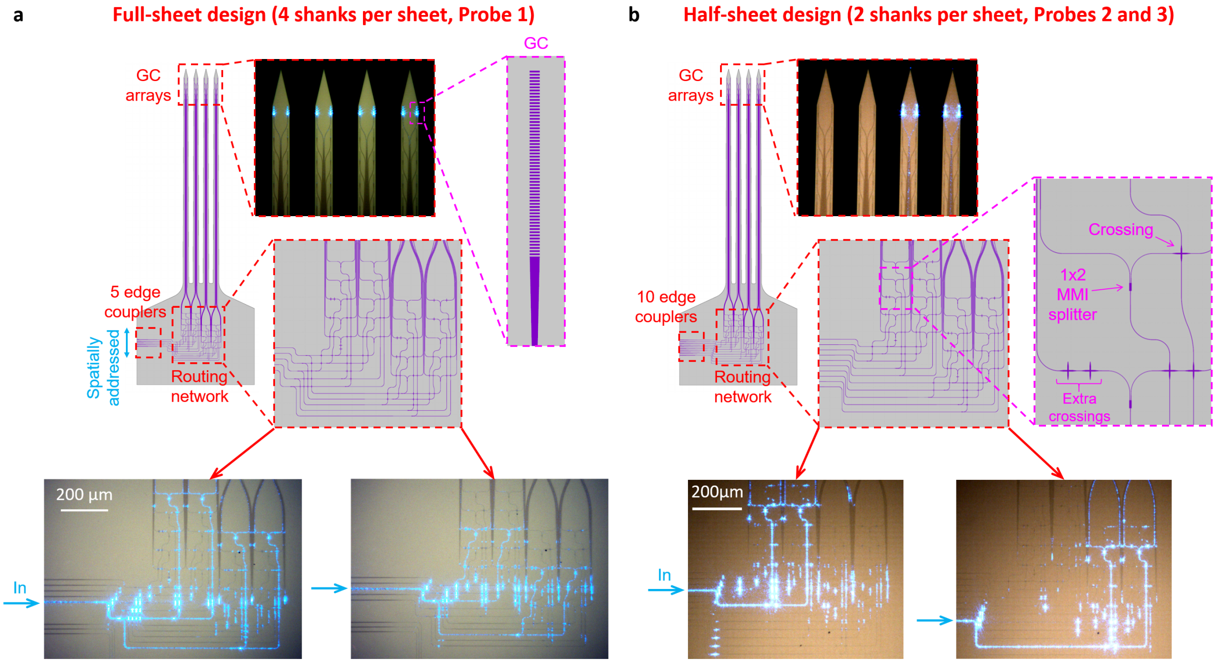

Figure S1: Additional details of the light-sheet neural probe designs. Comparison of the light-sheet neural probe designs with (a) 4 shanks per sheet and (b) 2 shanks per sheet. The design in (a) has 5 independent sheets and 5 edge couplers, and the design in (b) has 10 independent sheets and 10 edge couplers. Schematics of the probes with magnified views of the routing networks are shown. Microscope images of the grating couplers (GCs) emitting a single sheet are shown for both designs. Microscope images of the routing networks with optical inputs to 2 different edge couplers are also shown for both designs. The rightmost inset in (b) shows the 1 × 2 MMI power splitters and waveguide crossings used in the routing networks. Our design and characterization of these devices is described in [34]. For transverse-electric (TE) polarized light at a wavelength of 488 nm, the measured excess loss of the 1 × 2 MMI power splitters was ≈ 0.5 dB per power splitter, and the loss of the crossings was < 0.2 dB/crossing [34].

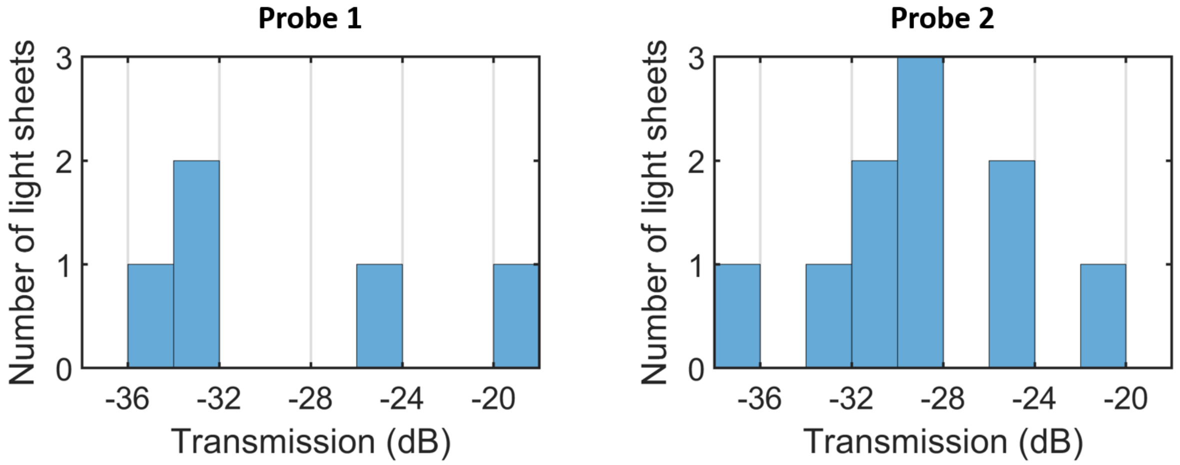

Figure S2: Histograms of the optical transmissions of the light sheets from packaged Probe 1 and Probe 2. The transmission is the ratio of the emitted power from the GCs to the input laser power to the scanning system.

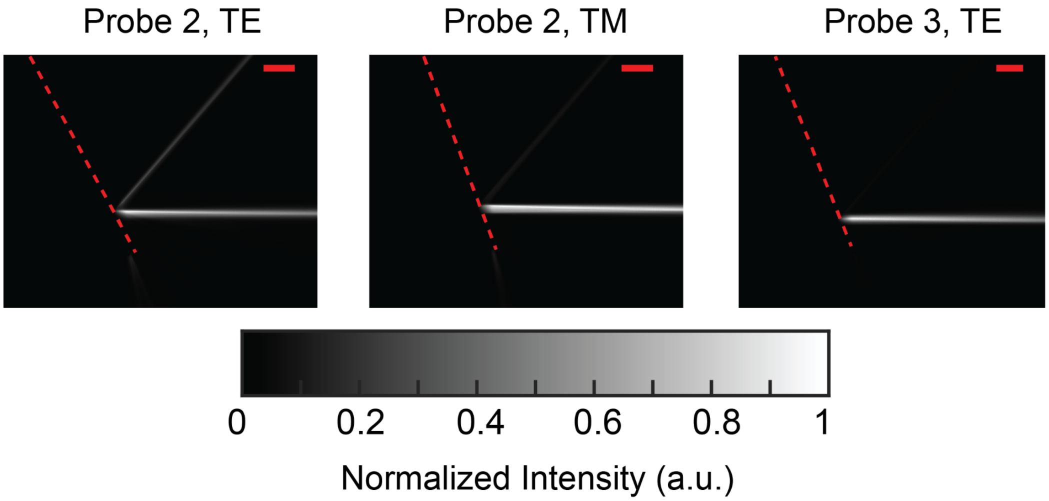

Figure S3: Additional fluorescence images of side sheet profiles from light-sheet neural probes immersed in fluorescein. Left: Probe 2 for the TE polarization. Center: Probe 2 for the TM polarization. Right: Probe 3 for the TE polarization. The dashed red lines delineate the top surface of the shanks. The scale bars are 100 μm.

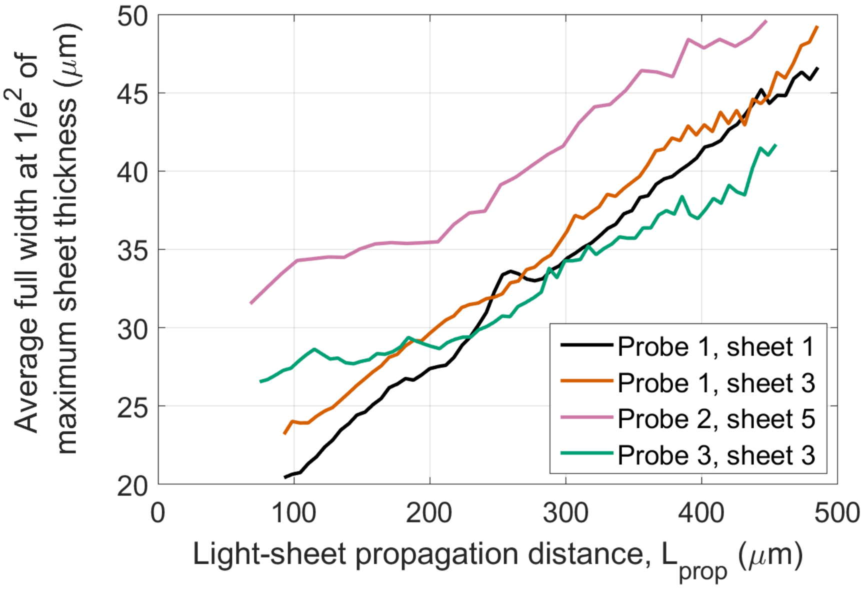

Figure S4: Additional data for the free-space light-sheet profile measurements in Fig. 4. Average full width at $1/e^2$ of maximum sheet thickness versus light-sheet propagation distance for sheets from Probes 1 – 3. The sheet thicknesses are averaged over the sheet width, the vertical axis in Fig. 4(c). The FWHM sheet thickness for Probe 1 – Sheet 2 is shown in Fig. 4(b), but the $1/e^2$ sheet thickness could not be measured accurately and is not shown here because the relatively weak transmission of the sheet resulted in $1/e^2$ intensity levels close to the noise floor of the measurement. In general, the $1/e^2$ sheet thicknesses increase linearly with propagation distance, while the Probes 2 and 3 FWHM sheet thicknesses in Fig. 4(b) are nonlinear with propagation distance, a result of the evolution of the sheet profiles with propagation distance.

Supplementary Note 1: Fixed tissue imaging data analysis

Neuron regions of interest (ROIs) were identified within the fixed tissue fluorescence images using the following algorithm. First, background subtraction was performed on the images. The background was estimated with a moving average window across the image. Next, adaptive thresholding followed by a morphology operation was performed on the image to extract regions with prominent peaks [47]. Local peak detection with watershed segmentation was then applied to each extracted neuron ROI for separating ROIs merged together due to close proximity. Manual intervention was done to reject false detection and to include undetected neurons. Neuron ROIs were defined manually using Fiji [48] to approximate closely the outlines of the neuron somas. In Fig. 6(a), 47 neuron ROIs were defined by the algorithm above and 22 neuron ROIs were defined manually. In Fig. 6(b), all ROIs were defined manually.

For the contrast calculation, dark-frame subtraction was performed for ambient illumination removal. The ambient level in the frame was estimated by averaging over 35 frames for Fig. 6(a) and 44 frames for Fig. 6(b) with the absence of excitation illumination. Then, each neuron mask was dilated by 5.7 μm (20 pixels) to form an annular region surrounding the neuron ROI. Next, we calculated the contrast of each neuron with the following equation:

$$contrast = \frac{F_{neuron} - F_{background}}{F_{background}} \tag{1}$$

Here, F_{neuron} is the mean fluorescence value within the ROI of the neuron mask and $F_{background}$ is the mean fluorescence level within the annular mask. Portions of the annular masks overlapping the ROIs of other neurons were excluded from the background calculation.

For the fixed tissue and *in vitro* imaging, image and statistical analyses and plotting were performed using MATLAB (MathWorks Inc., Natick, MA, USA) and Python (version 3.6.7) [49]. The maximum and minimum of the intensity scales for the grayscale fixed tissue fluorescence images in Fig. 6 were set as the maximum and minimum of all pixel values in the corresponding raw images.

Supplementary Note 2: *In vitro* imaging data analysis

First, any vibration in the calcium imaging video was corrected with the rigid motion correction method. Then, active neurons were detected using the constrained non-negative matrix factorization (CNMF) algorithm. Neurons with SNR below 2 and spatial correlation of the footprint during active phase below 0.85 were filtered to reduce the false detection rate. The neuron detection described above was performed with the analysis code provided in [50]. Next, manual inspection of the video was conducted to ensure the quality of the detected signals. For the GCaMP6s slice analysis, manual inspection of the time-dependent fluorescence traces was used to remove falsely detected neurons and to include undetected neurons; neuron ROIs were defined manually using Fiji. For the Cal-520 AM slice analysis, neuron ROIs were defined by the analysis code in [50], and manual inspection of the fluorescence change, $\Delta F/F$, traces was used to remove detected neurons with any of the following: 1) no detected peaks in $\Delta F/F > 0.5$ (using the Matlab *findpeaks* function), 2) $\Delta F/F$ noise levels high enough to be detected as peaks using the *findpeaks* function, 3) $\Delta F/F$ traces without characteristics consistent with Cal-520 AM calcium event dynamics.

$\Delta F/F$ traces were calculated as follows. For each identified active neuron, a binary mask was generated, and an annular region with a width of 6 μm surrounding the neuron ROI was also generated. The fluorescence of each neuron was spatially averaged over the neuron ROI, resulting in a time-dependent trace F_{raw} for each neuron. To partially correct for neuropil fluorescence and illumination fluctuations, background removal was performed on each F_{raw} with the background signal (F_{bg}) estimated by the first percentile of the fluorescence intensity within the annular region surrounding the neuron ROI [51, 52]. $F_{baseline}$ is the baseline of $F_{raw} - F_{bg}$, and for the Cal-520 AM slice analysis, $F_{baseline}$ was determined by the average of the lowest 10% of the values within a 7 s wide moving window swept over $F_{raw} - F_{bg}$ [53]. Moving windowing was not used for GCaMP6s slice data due to the long duration of some of the calcium events. Instead, $F_{baseline}$ was taken as the average of the lowest 10% of the whole $F_{raw} - F_{bg}$ time trace for a video segment (segments defined in Fig. 12). The fluorescence change, $\Delta F/F$, for each neuron was then calculated as:

$$\frac{\Delta F}{F} = \frac{F_{raw} - F_{bg} - F_{baseline}}{F_{baseline}} \, . \qquad (2)$$

A small number of ROIs with dimensions significantly smaller than the surrounding cells are present in the *in vitro* Cal-520 AM imaging analysis in Figs. 8(b) and (c). Such an ROI is an "island" of a larger adjacent ROI, both belonging to the same cell. These islands occur because the CNMF algorithm does not guarantee an enclosed shape for a neuron ROI [50]. The islands were not considered to be separate from their parent ROIs for the calculations. These islands did not occur in the analysis of the *in vitro* GCaMP6s brain slice imaging in Fig. 7 since the ROIs were defined manually.

The maximum and minimum of the intensity scale of each grayscale *in vitro* GCaMP6s brain slice image in Figs. 7, 11, and 12 were set as the maximum and minimum pixel intensity values of the raw fluorescence (or maximum projection fluorescence) image (using MATLAB). The *in vitro* Cal-520 AM grayscale maximum projection fluorescence images in Figs. 8, 13, and 14 were generated from raw video data using Fiji with the grayscales set using the *autoscale* function.

Videos 1 – 3 and S1 – S4 were processed using Fiji. To generate Videos 1 – 3 and S4, the raw 16-bit calcium imaging videos were cropped to the areas analyzed in Figs. 7 and 8, the grayscales (brightness and contrast) were adjusted, and the videos were then converted to mp4 video files (8-bit, h.264 compression). The procedure for setting the intensity grayscales of Videos 1 – 3 and S4 is as follows. For each video, the maximum pixel intensity of each frame was compiled into a distribution of maxima and the 99th percentile of the distribution was found. For each video, the 1st percentile of the distribution of minimum pixel intensities of all frames was also found. Due to the similarity of the maximum fluorescence intensities in the GCaMP6s imaging in Videos 1 and 2, the same intensity grayscale was applied to both videos; the maximum and minimum of the grayscale were set as the larger 99th percentile of maxima and the smaller 1st percentile of minima, respectively, of the two videos. The maximum fluorescence values for the Cal-520 AM imaging in Videos 3 and S4 differed significantly and required different intensity grayscales for each video. The maximum and minimum of each grayscale in Videos 3 and S4 were set as the 99th and 1st percentiles of the distributions of maxima and minima of each video, respectively.

Supplementary Note 3: *In vivo* imaging data analysis

The calculation of ΔF/F for the *in vivo* imaging data in Fig. 9 follows the calculation in Supplementary Note 2 but without background subtraction in the baseline fluorescence; ΔF/F in Fig. 9(c) also uses a 5 frame moving average and the annular ROI for background calculations is 14 μm wide.

Supplementary Note 4: Additional details of the neural probe used for *in vivo* imaging

The light-sheet neural probe used for the *in vivo* fluorescence imaging in Fig. 9 was a prototype that preceded our foundry fabricated probes. This probe was fabricated using the method described in [26] at the California Institute of Technology's Kavli Nanoscience Institute cleanrooms. Briefly, these probes were fabricated on 100 mm diameter silicon-on-insulator (SOI) wafers with a 15 μm thick Si device layer and a 2 μm thick buried oxide layer. A 1.5 μm thick thermally grown SiO_2 layer was used as the bottom cladding of the waveguides. A 200 nm thick silicon nitride (SiN) waveguide layer deposited by low pressure chemical vapor deposition (LPCVD) was patterned and etched by electron beam lithography and an inductively coupled plasma (ICP) pseudo-Bosch etch process. The waveguide top cladding was a 1 μm thick plasma enhanced chemical vapor deposition (PECVD) SiO_2 layer. Front- and back-side deep reactive ion etching (DRIE) was used to define the probe shape, edge coupler facets, and selectively thin the shanks to a thickness of ≈ 18 μm, while leaving the base of the probe chips (containing the routing network and edge couplers) at the full ≈ 300 μm thickness of the SOI wafer.

Other than the thickness, the designed dimensions of these neural probes were nearly identical to those of the foundry fabricated neural probes. The shank pitch was 135 μm, the shank length was 3 mm, the pitch of the rows of grating couplers (GCs) for each sheet was 80 μm, the GC socket width was 1 μm, and the GC period was 400 nm with a 50% nominal duty cycle.
